# Cortical Modulation of Motor Control Biofeedback among the Elderly with High Fall Risk during a Posture Perturbation Task with Augmented Reality

**DOI:** 10.3389/fnagi.2016.00080

**Published:** 2016-04-28

**Authors:** Chun-Ju Chang, Tsui-Fen Yang, Sai-Wei Yang, Jen-Suh Chern

**Affiliations:** ^1^Department of Biomedical Engineering, National Yang-Ming UniversityTaipei, Taiwan; ^2^Department of Physical Medicine and Rehabilitation, Taipei Veterans General HospitalTaipei, Taiwan; ^3^Department of Physical Therapy and Assistive Technology, National Yang-Ming UniversityTaipei, Taiwan; ^4^Graduate Institute of Rehabilitation Counseling, National Taiwan Normal UniversityTaipei, Taiwan

**Keywords:** cortical modulation, motor control, fall risk, computerized dynamic posturography, virtual reality, augmented reality

## Abstract

The cerebral cortex provides sensorimotor integration and coordination during motor control of daily functional activities. Power spectrum density based on electroencephalography (EEG) has been employed as an approach that allows an investigation of the spatial–temporal characteristics of neuromuscular modulation; however, the biofeedback mechanism associated with cortical activation during motor control remains unclear among elderly individuals. Thirty one community-dwelling elderly participants were divided into low fall-risk potential (LF) and high fall-risk potential (HF) groups based upon the results obtained from a receiver operating characteristic analysis of the ellipse area of the center of pressure. Electroencephalography (EEG) was performed while the participants stood on a 6-degree-of-freedom Stewart platform, which generated continuous perturbations and done either with or without the virtual reality scene. The present study showed that when there was visual stimulation and poor somatosensory coordination, a higher level of cortical response was activated in order to keep postural balance. The elderly participants in the LF group demonstrated a significant and strong correlation between postural-related cortical regions; however, the elderly individuals in the HF group did not show such a relationship. Moreover, we were able to clarify the roles of various brainwave bands functioning in motor control. Specifically, the gamma and beta bands in the parietal–occipital region facilitate the high-level cortical modulation and sensorimotor integration, whereas the theta band in the frontal–central region is responsible for mediating error detection during perceptual motor tasks. Finally, the alpha band is associated with processing visual challenges in the occipital lobe.With a variety of motor control demands, increment in brainwave band coordination is required to maintain postural stability. These investigations shed light on the cortical modulation of motor control among elderly participants with varying fall-risk potentials. The results suggest that, although elderly adults may be without neurological deficits, inefficient central modulation during challenging postural conditions could be an internal factor that contributes to the risk of fall. Furthermore, training that helps to improve coordinated sensorimotor integration may be a useful approach to reduce the risk of fall among elderly populations or when patients suffer from neurological deficits.

## Introduction

Motor control is the process of sensorimotor integration that is required when performing functional activities; this involves the coordination of various regions in the cerebral cortex. Among elderly individuals, aging, which is accompanied by a combination of degeneration of sensation pathways, limb functions and cognition, may result in inefficient postural actions and these can increase the risk of falling. The self-reporting falling experiences, functional assessments, and the integrated sensory test for balance have all been commonly used during fall-risk screening (Boulgarides et al., 2003; Muir et al., 2008); however, a ceiling effect has frequently been reported to be a weakness of using these assessments and this can lead to a miss-assessment of an individual’s risk of falling (Boulgarides et al., 2003; Pardasaney et al., 2012). Furthermore, these assessments are not capable of evaluating any underlying impairments that affect sensorimotor functionality and muscular synergy coordination, both of which are important rationales thought to be involved in causing the elderly to fall.

In order to carry out an effective assessment of the various types of sensory impairments that may be present in the elderly, computerized dynamic posturography (CDP) that uses multiple-direction perturbed motions has been widely adopted as a means of complex balance assessment. In combination with clinical evaluation protocols, this system provides not only an individual sensorimotor impairment diagnosis but also an assessment of the overall motor responses related to the postural balance (Brauer et al., 2002; Tsai et al., 2014). It has been shown that the CDP system is a superior fall-risk detection methodology than the static balance test, and this approach appears to be a more sensitive tool when identifying individuals who are at high-risk of recurrent falls (Buatois et al., 2006). When depicting postural stability, data derived from the center of pressure (COP) has been used to represent the effective stability of the center of mass (COM) during the postural control (Varghese et al., 2015), and this has been found to be a valid predictor of the risk of falling (Pajala et al., 2008). In particular, the ellipse area of the COP, which represents quantified temporal information with respect to postural stability (Baltich et al., 2014), and is considered to be an essential part in posturography study (Schubert and Kirchner, 2014). The results of CDP studies have shown that a larger postural sway and a longer reaction time are more often part of the compensatory strategy used for posture recovery among the elderly than among young adults (Brauer et al., 2002; Chang, 2009). Specifically, balance-impaired elderly individuals did not show any postural adaptation during the perturbed motion itself (Buatois et al., 2006).

The use of virtual reality (VR) system intensifies the involvement of patients in rehabilitation and this is due to the presence of a lively background as well as the fact that VR allows the close simulation of daily tasks (Yang et al., 2011). Furthermore, VR also makes it possible to incorporate additional methods of detecting underlying motor control deficits (Pardasaney et al., 2013). A VR-based balance assessment using a moving platform has previously shown that an interrupted visual interference will induce a significantly larger postural sway in young adults, as compared to a static scene, and the finding suggested to the authors a possible clinical application of this approach in the area of identifying postural control capacity deficits (Lee et al., 2004). Compared to vestibular information, visual and proprioceptive inputs play dominant roles in maintaining postural stability (Lee et al., 2004). Buatois et al. (2006) also concluded that multiple-fallers show significantly greater postural sway than non-fallers and single fallers when their vision is occluded, and this seems to indicate a greater reliance on visual information when maintaining postural stability among persons with postural control deficits (Tossavainen et al., 2003). The usage of CDP at the same time with VR-based scene is a novel assessment tool and is a form of augmented reality (AR), which provided not only a near-real visual effects but enhance the real-time reactions of the body segments to the external perturbations. There has been little research published exploring the mechanisms underlying cortical integration during motor control and posturography under AR conditions, and such an approach becomes potentially in this research area.

When ensuring a concordant signaling process from the sensory afferent system to the motor efferent system, a balance challenge task requires a high degree of sensorimotor integration with the rhythm of the body segments for maintaining stability (Jacobs et al., 2015; Varghese et al., 2015). The power spectrum density (PSD) of an EEG depicts the spatial–temporal characteristics of the cortical modulation that occurs in response to neuromuscular biofeedback (Petrofsky et al., 2012; Slobounov et al., 2013; Tse et al., 2013; Hülsdünker et al., 2015), and the changes in the brainwave bands in a specific cortical area has been shown to be associated with postural control. For example, activity in the anterior cingulate cortex and posterior parietal cortical regions has been shown to change during balance instability (Hülsdünker et al., 2015). In health subjects, the activation of the parietal and prefrontal cortex seems to increase as afferent visual signaling changes (Del Percio et al., 2007). Using brainwave band analysis, it has been shown that an increase in the theta band power of the frontal and parietal lobes reflects sensory information transfer and processing (Sipp et al., 2013). On the other hand, an increase in beta band power in the sensorimotor cortex is associated with muscle contraction (Jacobs et al., 2015). Furthermore, a burst in gamma wave amplitude has been found to process the initiation of compensatory backward movement when balance is threatened (Slobounov et al., 2005). It has also been suggested that an increase in gray matter volume is correlated with an improvement in balance ability (Taubert et al., 2010). All these findings support the proposed hypothesis that motor control involves cortical modulation to integrate the sensory feedback needed for voluntary postural adjustments, but not just a simple reflex response (Horak, 2006).

Several recent but limited studies have investigated neural modulation in a static position or during interruption of only one of the senses. These results have shown that, while performing common sensorimotor balance tasks that involve multisensory stimuli, such as standing with eyes closed, taking up a tandem stance (standing with one foot ahead the other) or standing on a foam mat, the power levels of the beta and sigma band are found to increase significantly within the central and parietal regions as compared to a natural stance with eyes open (Tse et al., 2013). Moreover, the amount of beta band corticomuscular coherence among young adults has been found to be significantly greater when taking on a wide stance with eyes open than taking on a narrow stance (Jacobs et al., 2015). Among young participants, theta power during an unpredicted perturbation task shows a stronger modulation in frontal–central regions than when the same assessment is carried out during a predictable task (Slobounov et al., 2013). Thus, while these studies have investigated how cortical modulation controls postural stability under static conditions, there have been no investigations to our knowledge targeting the mechanisms of cortical modulation during dynamic posture control.

Given that little is known about sensorimotor integration and neural correlations with motor control during the motor control processes, this study investigated cortical modulation mechanisms when elderly participants with varying levels of fall risk were subjected to the CDP combined with a VR based scene thus creating an AR environment. This paradigm allowed us, firstly, to gain insights into the cortical modulation of postural responses during visual interference and perturbation; this was done by comparing a low fall-risk elderly group and a high fall-risk elderly group. Secondly, we wished to gain an understanding of the role of wave band coordination among the various cortical regions that are responsible for maintaining postural stability. In this part of the study, we observed the presence of cortical modulation associated with motor control among the elderly. Finally, we make suggestions regarding the use of posturography, coupled with AR training for enhancing cortical modulation by patients with neurological deficits and among those who are at an increased risk of falling, particularly when there are challenges that affect postural control.

## Materials and Methods

### Participants

A total of 31 community-dwelling elderly participants were recruited. The inclusion criteria were that they had the ability to walk and climb stairs without assistive devices and had no known pathological condition that could impair balance. Elderly participants who had been diagnosed with any cardiac, pulmonary, visual, or vestibular impairment, any neuromusculoskeletal disorder, or a cognitive deficit were excluded.

Based on previous studies, the variable of COP ellipse area during VR-based posturography balance-maintaining movement was considered to be the critical measure of postural movement controlled by the central nerve system (Lee et al., 2004; Chang, 2009), and this was used to discriminate the fall-risk potential among the elderly subjects (Buatois et al., 2006; Macedo et al., 2015). The optimal cutoff values for the COP ellipse area with respect to postural instability (Knapp et al., 2011; Masedu et al., 2013) was determined by the receiver operating characteristic methodology (Schisterman et al., 2005). Individuals with an COP ellipse sway area value less than 7.63 cm^2^ during VR-based posturography balance-maintaining movement were identified as belonging to the low fall-risk group (LF), while those individuals whose value exceeded 12.48 cm^2^ were identified as belonging to the high fall-risk group (HF). The detailed methodology used in this study is presented in Supplementary Table S1.

Fifteen elderly participants were placed in the LF group (5 males, 10 females, age 68.4 ± 2.58 y/o, height 158.3 ± 9.2 cm, weight 62.4 ± 11.2 kg), while 16 elderly participants were placed in the HF group (7 males, 9 females, age 70.2 ± 2.2 y/o, height 161.0 ± 8.2 cm, weight 64.8 ± 8.6 kg). There was no significant difference in demographic data between the two groups. However, there was a significant difference in the COP ellipse area between the two groups (LF: 5.0 ± 3.1 cm^2^, HF: 28.9 ± 13.8 cm^2^, *p*= 0.000) as determined by the independent *t*-test. Each participant provided written informed consent and the study was approved by the Institutional Review Board of Taipei Veterans General Hospital (No. 2014-01-003C).

### Procedures

All participants were required to maintain stability as much as possible while standing barefoot on a moving platform and wearing a full-body harness to prevent falling. The EEG data was recorded during the perturbation; this was either synchronized with or without a VR passenger bus driving over potholes in a random manner. No practice trial was allowed in order to avoid any learning effect, but rests were permitted as required. To enhance the EEG signal properties, the participants were asked to clean their scalp as thoroughly as possible before coming to the laboratory. A standard preparation procedure based on the requirements of the instrument (Pivik et al., 1993) was followed before the EEG electrodes were applied to the participant.

### Instruments

A 6-degree-of-freedom (DOF) Stewart platform (DOF Technology, Taiwan), equipped with a customized and computerized perturbation protocol, was used as the main moving platform in this study. The CDP coupled with the VR-based system has been used previously to induce significant postural responses and has been found to be sufficiently sensitive for identification of participants with an increased fall-risk (Chang, 2009). There was a need for the multiple-direction perturbations that were designed to allow the detection of somatosensory and vestibular sensory integration in motor control ability rather than as simple translation or rotational movements (Tsai et al., 2014); therefore, the composition of forward–backward motion and pitching motion were used in this study.

**FIGURE 1:**
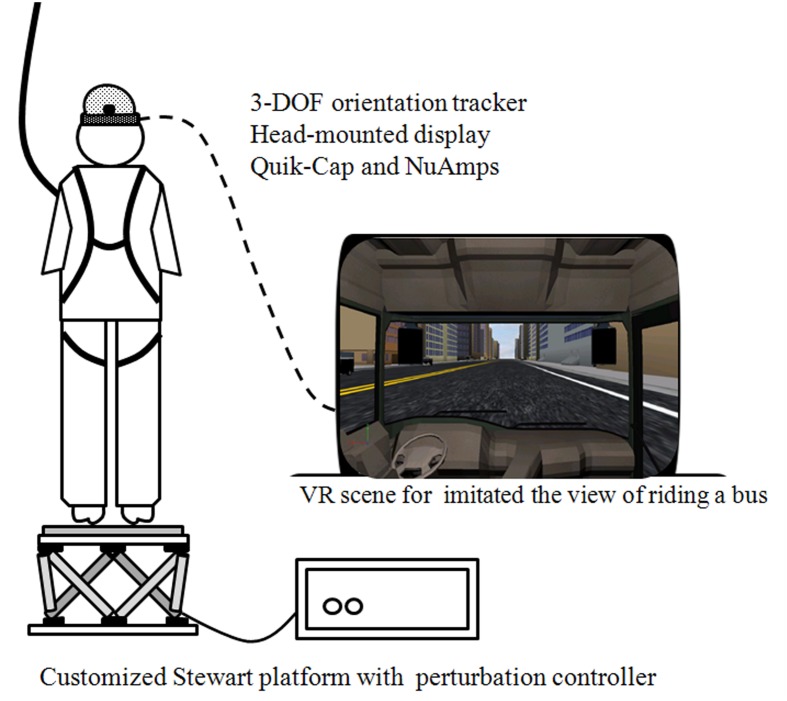
**Instrument and virtual reality set-up.** Each participant was required to stand barefoot on a Stewart platform that induced continuous perturbed motions, and was either synchronized with or without the VR-based scenery which represented the view of riding on the passenger bus. The electroencephalography signals were also recorded synchronously and a full-body harness was worn to prevent falling.

The protocol contained four continuous and sequential motion phases, which can be described as follows: (0) the stationary (ST) phase, in which the platform was stationary, and lasted for 5–10 s randomly presented to participant in order to prevent any anticipated effect; (1) the slip-forward (SF) phase, in which the platform slipped forward from 0 to 11 cm in 1 s; (2) the pitch-down (PD) phase, in which the platform pitched down from 0 to 10°, which was accompanied by a simultaneous forward translation from 11 to 14 cm in 0.5 s; (3) the pitch-up (PU) phase, in which the platform pitched up from the end point of the PD phase, which was accompanied by a simultaneous backward translation from 14 to 11 cm in 0.5 s; and (4) the recovery (RE) phase, in which the platform recovered from the end-point (the PU phase) back to the start-point (the ST phase) within 15 s (Chang, 2009; Tsai et al., 2014). The VR-based scenery (Virtools Dev 4.0) represented an individual’s view while riding on a passenger bus and had the platform moved in synchrony, which provided the directive interaction between environment and body segments. The scenery was projected via a head-mounted display (HMZ-T3W, SONY, Tokyo, Japan) that was worn with a 3-DOF orientation tracker (InertiaCube4^™^, InterSense, Billerica, MA, USA) in order to allow enhanced the augmentation (**Figure 1**). The Stewart platform and the head-mounted VR system with 3-DOF orientation tracker formed the AR system used in this study. The VR alone usually represents the visual effect only and the moving platform is used mainly for the somatosensory system dysfunction evaluation. The integration of the VR system and the Stewart platform created a novel AR environment compared to when the two systems were separated.

The EEG dataset was recorded continuously using a 32-channel Quik-Cap (Compumedics Neuroscan, Charlotte, NC, USA) designed according to the International 10–20 electrode system. Reference electrodes were placed on each side of the mastoid process (A1/A2), and the ground electrode was placed on the forehead. A NuAmps amplifier and SCAN4.3 software (Compumedics Neuroscan) were used for recording at a sampling rate of 1000 Hz; the impedance of the Ag/AgCl electrodes were maintained below 5 kΩ, while the bandpass filter DC was set at 70 Hz, the notch filter was set at 60 Hz, and the gain was set at 19 during signal processing (Thibault et al., 2014; Hülsdünker et al., 2015; Jacobs et al., 2015; Varghese et al., 2015). All instruments were integrated and synchronized by the AD controller of the Steward platform.

### Data Processing and Analysis

The EEG dataset was processed using EEGLAB toolbox (Delorme and Makeig, 2004), which operated under a MATLAB R2012a environment. Variables of the PSD were calculated by setting the bandpass filter at 0.1–50 Hz, using the Welch method in 128 windows with 50 overlaps (Petrofsky et al., 2012; Varghese et al., 2015), which allowed estimation of the absolute PSD for each wave band (theta: 4–7 Hz, alpha: 8–12 Hz, beta: 12–30 Hz, gamma: 30–40 Hz). To reduce individual variation, the relative PSD in each motion phase was acquired by dividing the absolute PSD during the various motion phases by the absolute PSD during the ST phase (Tse et al., 2013) and subsequently the boxplot function of MATLAB was employed to exclude all outliers.

$$\text{relative PSD =}\frac{\text{absolute PSD in SF, PD, PU, RE phase}}{\text{absolute PSD in ST phase}}$$

### Statistical Analysis

SPSS 21.0 software was used for the statistical analysis. The Shapiro–Wilks test was first used to assess whether the data was normally distributed. The interaction between vision (noVR vs. VR) × group (LF vs. HF) factors was analyzed by two-way repeated measures ANOVA. The degree-of-freedom was adjusted using the Mauchly test for sphericity assumption, and Greenhouse–Geisser adjustment was used to correct for this. Pearson’s correlation coefficient was employed to analyze the correlation between relative PSD across the posture-related cortex. A *p*-value < 0.05 was considered to be significant.

## Results

### Relative Power Spectrum Density at Each Platform Phase and for Each Band

**FIGURE 2:**
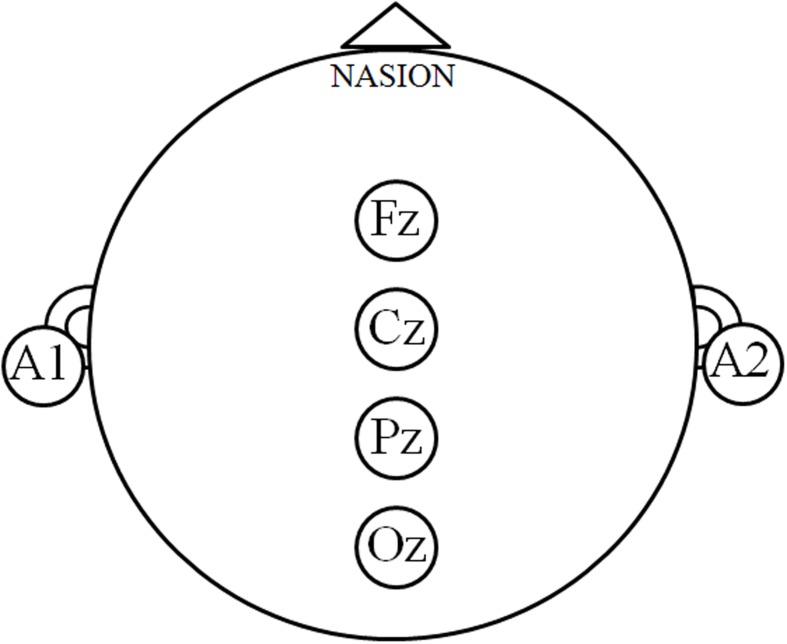
**Posture-related cortex regions.** The electroencephalography signals at the Fz, Cz, Pz, and Oz sites denote the posture-related cortical regions, and the relative power spectrum density (PSD) was calculated in order to describe the cortical modulations during motor control.

The interaction between group and vision factors was analyzed first, and then the main effect of the group or vision factor was then tested. Only data from the Fz, Cz, Pz, and Oz sites were analyzed in order to target the posture-related cortical areas that are involved in motor control (**Figure 2**; Slobounov et al., 2005, 2013; Tse et al., 2013; Hülsdünker et al., 2015).

**FIGURE 3:**
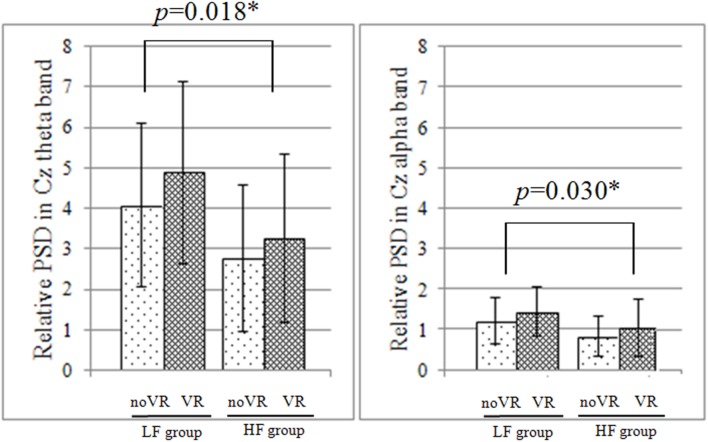
**Relative PSD at Cz during the initial platform slip forward phase.** In both the theta and alpha band, there was no significant interaction between vision × group factors, but there was a significant simple main effect of the group factor based on the data from Cz; furthermore, the low fall-risk potential (LF) group had significantly higher power spectrum density (PSD) than the high fall-risk potential (HF) group.

Neither the Fz nor Cz sites exhibited any significant interaction between vision × group factors (*p* > 0.05), but a main effect was still noted for both. A main effect of the vision factor was observed at Fz, with the VR resulting in significantly higher PSD during the PU (noVR: 0.001 - 0.256, VR: 0.001 - 0.354, *p*= 0.002 - 0.022) and RE (noVR: 4.8 - 5.5, VR: 6.0 - 6.9, *p*= 0.026 - 0.030) phases. This was also true at Cz during the PU (noVR: 0.001 - 0.305, VR: 0.001 - 0.443, *p*= 0.003 - 0.014) phase. The main effect of the group factor was observed at Cz, with a higher PSD in the LF group than in the HF group for the SF phase theta band (LF: 4.0 - 4.8, HF: 2.7 - 3.2, *p*= 0.018) and alpha band (LF: 1.2 - 1.4, HF: 0.8 - 1.0, *p*= 0.030; **Figure 3**).

**FIGURE 4:**
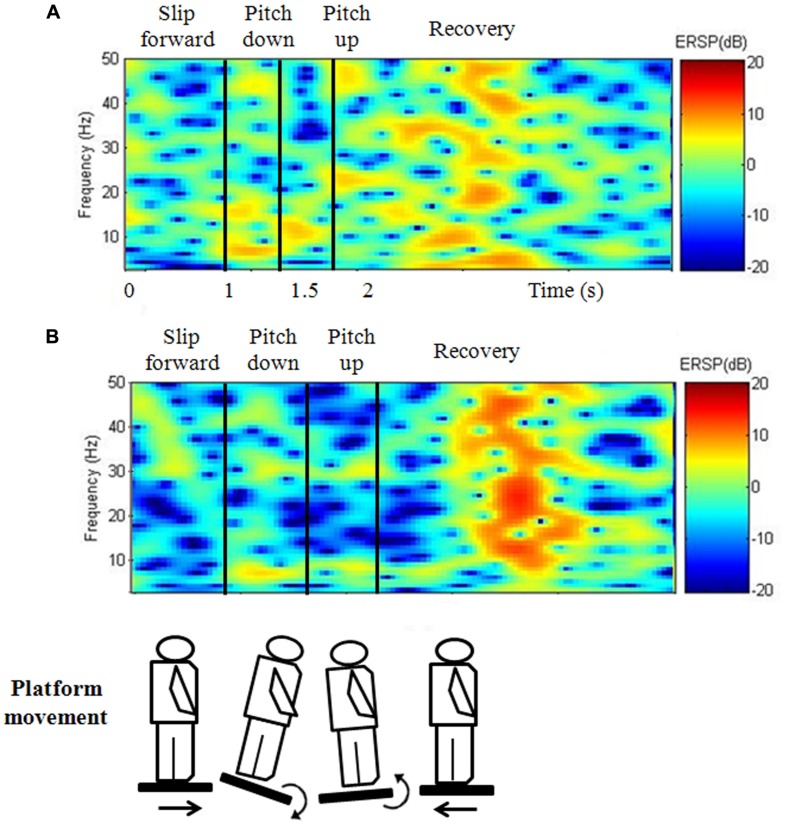
**Frequency–time domain at Pz during the continuous perturbed motion with virtual reality for the (A) LF and (B) HF groups.** During the platform recovery phase at parietal region, the HF group demonstrated a significantly greater cortical activation than the LF group for compensating postural instability.

Pz and Oz demonstrated a significant interaction (*p*= 0.030 - 0.031, *p*= 0.034 - 0.042) between vision × group factors during the RE phase (**Table 1**). Specifically, at Pz, a simple main effect of the vision factor was observed in that the VR results showed a significantly higher PSD than under the noVR condition (*p*= 0.010 - 0.011); furthermore, a simple main effect of the group factor was also found with the elderly subjects in HF group demonstrating a significantly higher PSD than the elderly subjects in the LF group (*p*= 0.049). In the figure showing the frequency–time domain (**Figure 4**), the HF group showed significantly greater cortical activation at the parietal site than the LF group for all wavebands, especially during the platform recovery phase. At Oz, a simple main effect of the vision factor showed a significantly higher PSD under the VR condition than under the noVR condition during all phases (*p*= 0.005).

**Table 1:** Results of the relative power spectrum density (PSD) analysis by two-way repeated measure ANOVA of the Pz and Oz sites during the platform recovery phase with or without virtual reality.

Band	LF group		HF group		*p-*value		
	noVR	VR	noVR	VR	Vision × group	Vision	Group
***Pz site***							
Theta	5.1 ± 2.7	5.7 ± 4.0	5.4 ± 3.0	12.8 ± 9.7	0.031^∗^	0.011^∗^	0.049^∗^
Alpha	5.1 ± 2.7	5.8 ± 4.0	5.4 ± 3.0	12.9 ± 9.8	0.031^∗^	0.011^∗^	0.049^∗^
Beta	5.2 ± 2.2	6.0 ± 4.1	5.5 ± 3.2	13.3 ± 10.2	0.031^∗^	0.011^∗^	0.049^∗^
Gamma	5.6 ± 2.9	6.4 ± 4.4	5.8 ± 3.4	14.3 ± 11.1	0.030^∗^	0.010^∗^	0.049^∗^
**Oz site**							
Theta	4.7 ± 3.8	5.8 ± 5.6	3.8 ± 2.9	9.9 ± 6.1	0.042^∗^	0.005^∗∗^	0.384
Alpha	4.7 ± 3.8	5.8 ± 5.6	3.7 ± 2.9	9.9 ± 6.1	0.041^∗^	0.005^∗∗^	0.386
Beta	4.8 ± 4.0	6.0 ± 5.8	3.8 ± 3.1	10.3 ± 6.4	0.039^∗^	0.005^∗∗^	0.377
Gamma	5.2 ± 4.2	6.3 ± 6.1	4.1 ± 3.2	11.0 ± 6.9	0.034^∗^	0.005^∗∗^	0.360

*p < 0.05^∗^; p < 0.01^∗∗^; LF, low fall-risk potential group; HF, high fall-risk potential group.*

The gamma band showed a significant vision effect at Fz and Oz during every phase (noVR: 0.001 - 5.532, VR: 0.001 - 11.050, *p*= 0.000 - 0.043), and at Pz during the PU and RE phases (noVR: 0.001 - 5.637, VR: 0.002 - 14.399, *p*= 0.010 - 0.016). Moreover, the beta and theta bands displayed a significant vision effect at Fz and Oz (noVR: 0.01 - 5.14, VR: 0.01 - 10.30, *p*= 0.000 - 0.029), while the alpha band demonstrated a similar effect at Oz (noVR: 0.07 - 4.75, VR: 0.20 - 9.95, *p*= 0.000 - 0.005). All wave bands displayed a group effect during the RE phase at Pz, the HF group demonstrated a significantly higher PSD (HF: 5.4 - 14.3, LF: 5.1 - 6.4, *p*= 0.049), but only the theta and alpha bands demonstrated a group effect during the SF phase at Oz, while the LF group evoked a significantly higher PSD (LF: 1.2 - 4.8, HF: 0.8 - 3.7, *p*= 0.018 - 0.030).

### Correlation of Relative Power Spectrum Density between Posture-related Cortical Regions

**FIGURE 5:**
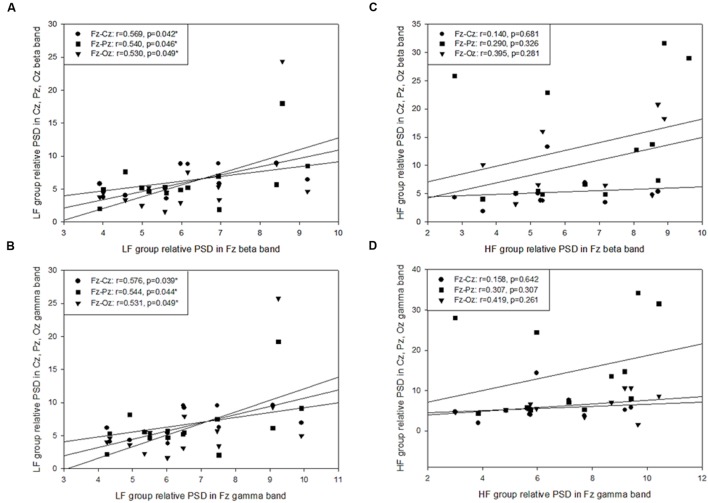
**Pearson’s correlation coefficient analysis of the posture-related cortex regions during the platform recovery phase with virtual reality interference.** The LF group demonstrated a significant correlation at Fz site and other regions in **(A)** the beta band and **(B)** the gamma band; however, there was no relationship found during the platform recovery phase among the HF group **(C,D)**.

On the basis of previous study (Chang, 2009), VR-based posturography backward recovery movement is considered to be the balance-maintaining movement after the perturbations, and is thought to reflect the capacity of central nerve system in terms of motor control. Therefore, Pearson’s correlation coefficient was used for investigating the cortical relationship between postural-related cortex regions. The results demonstrated that HF group showed a significantly moderate to high correlation among all regions for each band during the SF (*r*= 0.517 - 0.831, *p*= 0.000 - 0.048), PD (*r*= 0.587 - 0.938, *p*= 0.000 - 0.047), and PU phases (*r*= 0.643 - 0.927, *p*= 0.000 - 0.022), but there was no such correlation during the VR-based RE phase (*p* > 0.05; **Table 2**). The LF group displayed a significant correlation during the SF, PD, and PU phases, with significantly moderate correlations at the Fz - Cz site (*r*= 0.677 - 0.924, *p*= 0.000 - 0.016), Fz - Pz site (*r*= 0.673 - 0.910, *p*= 0.000 - 0.018), Cz–Oz site (*r*= 0.566 - 0.707, *p*= 0.007 - 0.044), and Cz–Pz site (*r*= 0.575 - 0.723, *p*= 0.005 - 0.031); furthermore, there were significantly high correlations at Fz (*r*= 0.529 - 0.576, *p*= 0.039 - 0.049; **Figure 5**) and Pz–Oz (*r*= 0.858, *p*= 0.000) sites during the VR-based RE phase (**Table 2**).

**Table 2:** Pearson’s correlation coefficient analysis of the PSD between posture-related cortex regions during the platform recovery phase with virtual reality interference.

Group		LF group			HF group		
Band	Site	Fz	Cz	Pz	Fz	Cz	Pz
Theta	Cz	0.566^∗^	–	–	0.131	–	–
	Pz	0.538^∗^	0.147	–	0.290	0.538	–
	Oz	0.529	0.538	0.858^∗∗^	0.396	–0.121	0.390
Alpha	Cz	0.566^∗^	–	–	0.132	–	
	Pz	0.539^∗^	0.147	–	0.291	0.538	–
	Oz	0.529^∗^	0.538	0.858^∗∗^	0.397	–0.118	0.390
Beta	Cz	0.569^∗^	–	–	0.140	–	–
	Pz	0.540^∗^	0.148	–	0.296	0.538	–
	Oz	0.530^∗^	0.534	0.858^∗∗^	0.404	–0.100	0.394
Gamma	Cz	0.576^∗^	–	–	0.158	–	–
	Pz	0.544^∗^	0.152	–	0.307	0.537	–
	Oz	0.543^∗^	0.525	0.858^∗∗^	0.419	–0.062	0.402

*p < 0.05^∗^; p < 0.01^∗∗^; LF, low fall-risk potential group; HF, high fall-risk potential group.*

## Discussion

This is the first study to our knowledge that has investigated the cortical modulations that are associated with postural responses using the AR and in elderly population. Below we discuss the main findings obtained from the relative PSD values and their correlation with postural-related cortical regions in order to clarify the mechanisms underlying variation in fall-risk potential among elderly individuals.

### Vision Effect of Relative PSD on Motor Control

The vision effect induced a higher PSD in the presence of VR, which supports our first paradigm; this was particularly obvious during the PU and RE phases for all posture-related cortical regions. As we expected, the more a postural task involves sensory interruption, the greater cortical activation involves in modulating the motor control biofeedback. This result is in line with the findings of other EEG studies where the beta and gamma bands in the frontal and occipital regions were found to increase more when individuals assumed a standing position compared to when they were in a sitting or lying position and that the beta band in the dorsofrontal and parieto-occipital regions is increased when the subject’s eyes are closed (Tse et al., 2013; Thibault et al., 2014; Slobounov et al., 2015). When sensation changes take place during balancing, such as standing on a foam-rubber surface, with narrow stance and with eyes closed, the beta and sigma bands have been found to increase significantly in the central-parietal area (Tse et al., 2013). The same modulation has been found in the athletes; specifically, athletes were found to have greater amplitude at ventral centro-parietal sites during the eye-closed Romberg test than in the eye-open condition (Del Percio et al., 2007). These findings confirmed that, when person is not able to rely on visual information for maintaining stability, neurological activity in regions of the prefrontal lobe, the visual-association cortex (Ouchi et al., 1999; Slobounov et al., 2005; Tse et al., 2013), and the parietal lobe are increased to allow processing to control postural stability at a higher level and to allow integration with visual demands (Mizelle et al., 2010). The more that reliance is placed on visual flow, the greater postural instability is observed when the sensory input is interrupted (Tossavainen et al., 2003). Moreover, as visual interference occurs, regions in the frontal, central, and parietal cortex are excited to allow sensorimotor integration and maintenance of postural stability. This confirms previous cortical visual processing studies, which suggested that the dorsal stream pathway is to coordinate the visual-motor biofeedback of the spatial information in order to overcome external interruptions. Therefore, to guide the motor actions in space, the neural pathway activates from the primary visual cortex in the occipital lobe and toward the parietal lobe (Ungerleider and Haxby, 1994; Milner and Goodale, 2008). Without the real visual guidance provided by the individual’s surrounding environment when there is VR interference in present study, the participant needs to utilize a great amount of cortical power within the occipital and parietal lobes in order to compensate for the presence of any unbalance posture and to enhance the coordination between the disturbed visual information and the alignment of the body.

Posture-related cortex activation was also found to be affected by VR-based interruption during the PU phase. The pitching motion required feedback based on the direction of the individual’s head for the purpose of navigation during motor control; however, the contribution of the vestibular system to the posture modulation process is not fully understood (Yoder and Taube, 2014), with the process seeming to be more complex than simply a involvement of the vestibular signal (Best et al., 2010). A few studies have proposed that the superior temporal gyrus or parietal–temporal junction plays a crucial role in processing vestibular information related to perception, ocular motion, and motor control (Best et al., 2010; Ventre-Dominey, 2014); the middle superior temporal region has been associated with visual tracking and has been found to contribute to self-induced or external motion in animal studies involving cats and monkeys (Grüsser et al., 1990); and the vestibulospinal tract is part of the vestibular system that coordinated the head and eye movements, and is responsible for the postural balance and head stabilization in some degrees (Lacquaniti et al., 2013). However, the temporal regions in present study did not show significant difference between the noVR and the VR-based conditions, it might due to the platform pitching perturbation was not large enough to produce a significant head oscillation to induce the vestibular response. Therefore, to maintain stability under the AR condition, elderly in both groups increased their PSD in order to bring into action a higher level of visual processing and sensorimotor integration that involves the frontal–central, central–parietal, and occipital lobes (Ouchi et al., 1999; Slobounov et al., 2005; Mizelle et al., 2010; Tse et al., 2013). The results of pitching motion in our study are also consistent with those of a study that employed static standing with a raw–pitch–yaw direction; under this condition the theta band power was significantly greater in the frontal–central regions (Slobounov et al., 2015), which is known to modulate error monitoring during motor performance (Adkin et al., 2006) and perceptual motor tasks (Ventre-Dominey, 2014).

### Effect of Differences in Band Frequency on Motor Control

For clarifying the function of the various brainwave bands in a range of body movements, this study analyzed brainwave bands with different frequencies during each platform movement phase. The gamma band displayed a strong vision effect at the frontal and occipital lobes during all phases and at the parietal regions during the PU and RE phases. This result is evidence of the involvement of conscious attention (Desai et al., 2015), especially during a challenging task, for example the tandem Romberg stance (Varghese et al., 2015). An increased excitation level was also identified, which suggests that there is central modulation controlling the postural actions needed to prevent falls (Slobounov et al., 2005). In the present study, the gamma band was active during all phases and it is likely to be involved in the processing of sensory feedback, which is consistent with Slobounov et al. (2005) results and is also evidence of conscious control of postural actions in elderly adults, specifically when there is a need to compensate for backward movements in response to postural perturbation.

The beta band showed a significant vision effect at frontal and occipital area, which is in line with Thibault et al. (2014) findings, namely that beta band activation increases when participants require greater control of vision and head orientation, this occurs in an 45° inclined position compared to a supine position. Both Thibault and our results supported the hypothesis that the beta band represents conscious concentration modulation throughout the motor cortex, such as when isotonic contraction is required (Desai et al., 2015; Jacobs et al., 2015). Furthermore, our results indicated that elderly participants needed to use higher levels of conscious attention when the motor cortex is modulating postural actions in response to novel postural perturbations (Ouchi et al., 1999; Slobounov et al., 2005; Mizelle et al., 2010); this involves a heightened state of awareness during the novel and unpredictable perturbation associated with an initial forward motion (Desai et al., 2015).

Slobounov et al. (2013) postulated that the theta band in the frontal–central area under unpredictable vision conditions reflects the assignment of brain resources to the completion of postural tasks. Other studies have also hypothesized that theta power in frontal–central regions may be related to the central mechanism of motor control (Gramann et al., 2006; Hülsdünker et al., 2015), to error detection (Hülsdünker et al., 2015), and to the execution of perceptual-motor tasks (Slobounov et al., 2005). Elderly participants with a high fall potential in our study were found to have the same neural reaction when they had to manage the greater vision interference present during the AR context, and the theta activation was observed to increase in the frontal and central areas, which are known to be associated with motor control and perceptual error detection. This result supports our paradigm that the incidence of falling in elderly might be caused by changes in the central mechanisms used for motor control.

The alpha band originates from the occipital lobe and thalamic regions and is correlated with cognitive performance and memory accuracy (Klimesch et al., 1993). Our findings demonstrate that a vision effect can be observed in the alpha band at the occipital lobe, suggesting that the person is temporarily idle, but is staying alert (Desai et al., 2015), and is prepared for the subsequent novel postural challenges. We, therefore, hypothesize that the elderly are still capable of reacting to novel events and should be able to learn the skills needed to manage challenging physical situations.

Frequency bands are able to elucidate the cortical mechanisms associated with modulating motor control, which links to our second purpose, namely that gamma and beta bands in the parietal–occipital region facilitate high-level cortical modulation and sensorimotor integration, whereas the theta band in the frontal–central region seems to be responsible for mediating error detection during perceptual motor tasks. Finally, the alpha band seems to be associated with processing visual challenges within the occipital lobe. When various motor control demands are present, increases in brainwave band coordination are required in order to maintain postural stability.

### Group Effect of Relative PSD on Motor Control

Only a few studies have focused on the effect of aging on cortical modulation during motor control, or on the risk of falling at the cortex level. As stated earlier, significant and diverse cortex modulation was observed in the frontal–central region and in the parietal–occipital region during the visual interference and postural perturbed task; furthermore, fall-risk potential was also found to be an important factor that caused distinct different cortical modulations when the two groups of elderly subjects were compared. The elderly individuals in the HF group displayed a higher level of cortical activity in the parietal lobe during the platform backward balance-maintaining phase, and their postural sway and cortical activation was increased when the situation involved a disruption of their sensory input, which indicates a diminution of sensorimotor integration during sensory input disruption. This result is consistent with a previous study in which patients were analyzed while in the tandem stance with eyes closed (Petrofsky et al., 2012). It was found that subjects with diabetes had higher levels in the beta and sigma bands of the parietal areas as well as greater postural sway than either age-matched participants without diabetes or young participants when the postural challenge was high and sensory feedback was occluded. It is reasonable to hypothesize that elderly individuals with a high risk of falling, although having no explicit neurological deficits, show inefficient central management under challenging postural conditions and this lack of management might be an internal factor that contributes to the risk of fall for this population. Nevertheless, further studies are needed to identify the neurological pathways involved.

In addition to the above findings, it was found that elderly individuals in the LF group demonstrated higher levels of cortical activity than the HF group in the central region across the theta and alpha bands during the initial platform slip forward phase. These findings can be related to those of Del Percio et al. (2007) where elderly individuals with a low fall-risk potential were found to possess the ability to maintain postural stability both centrally and peripherally and show greater alpha band amplitude; this is similar to the association found when subjects who were athletes specializing in karate were compared to subjects who did not carry out exercise regularly (Del Percio et al., 2007). The result of the findings on elderly individuals with a low risk of falling suggest that it might be necessary to training patients with the aim of improving their coordinated sensorimotor integration in order to reduce the risk of falling.

### Correlated Cortical Modulation in the Postural-related Cortex

In order to maintain stability, it is necessary for the various regions of the cerebral cortex to take part in the process of sensorimotor integration and this then creates adaptive postural responses. Among the functional areas of the human cerebral cortex, the prefrontal lobe is known to be involved in concentration and voluntary activities (Ouchi et al., 1999; Slobounov et al., 2005; Tse et al., 2013), while the supplementary and premotor cortical regions are known to modulate movement initiation and complex motion coordination (Slobounov et al., 2005); on the other hand the parietal lobe is known to mediate the sensorimotor integration (Mizelle et al., 2010; Hülsdünker et al., 2015; Jacobs et al., 2015; Varghese et al., 2015), while the visual association cortex is associated with high-level processing (Slobounov et al., 2005; Desai et al., 2015; Hülsdünker et al., 2015). The present study has demonstrated that there is a significant and strong correlation between the posture-related cortical regions in the LF group, but not in the HF group, during the platform recovery phase with visual interference. We can deduce from this that the LF group shows a great capacity for sensorimotor integration as well as better coordination in proprioceptive and visual information when reacting to complex postural responses, and one result of this was a decrement in postural sway during AR experiments. However, the HF group showed no correlation between these regions while the most challenging task in this study was examined; this diminished integration may be related to the significant postural instability of the subjects during the postural recovery phase when vision has been interrupted. In terms of the theory of the dorsal stream pathway (Milner and Goodale, 2008), the occipital lobe, parietal lobe and the sensorimotor cortex regions are connected in order to coordinate the spatial relationship between visual information and body alignments. Thus the LF group elderly in our study would seem to show a better correlation between above mentioned areas while maintaining stability. During our investigation of sensorimotor integration, we have clearly shown that elderly individuals with low and high fall-risk potentials use different degrees of cortical modulation during postural adaptation. Based on these findings, it is clear that a combination of AR and EEG is able to identify the neural correlates associated with motor control and cortical modulation, and this could have clinical applications during the rehabilitation of elderly populations and neurological patients whose are risk of falling.

The effective coordination of motor control requires the integrated and organized modulation of the sensory and neuromusculoskeletal systems. The somatosensory information ascends through the medial lemniscal pathway or spinocerebellar tracts and then relays to the cerebral cortex (Fling et al., 2014). For the motor function control, the pathway of pyramidal tracts transfers the motor neuron from the cerebral cortex and terminates either in the brainstem (corticobulbar tract) or spinal cord (corticospinal tract), and then activates the lateral or ventromedial system to manipulate the body’s muscles (Remaud et al., 2014). To maintain the upright postural stability, the biofeedback of neuro control pathway is through the spinal tract and subcortical structures such as basal ganglia, cerebellum and brainstem; in this study, we confirm that the voluntary motor movement is under the conscious control by the pathway of connections between cerebral cortex and the various subcortical structures, however, the cerebral cortex gray matter plays the role of sensorimotor integration and motor control modulation. The application of AR system in present study, not only induces near-real postural responses in a laboratory-based study, but also has helped to elucidate the mechanism of cortical modulation during motor control among elderly individuals. The measurement of brainwave bands at specific frequencies in various cortical regions that are dedicated to motor control has provided information on cortical modulation during various postural responses. Further studies on a population with a wider age range are necessary in order to determine the mechanism underlying the degeneration of motor control in the elderly, and to investigate the correlations between cortical activation, muscle activity and joint motion.

## Conclusion

The combination of AR with cortex power spectrum measure ment has provided novel insights into cortical modulation during motor control. In present study, we confirm that the more a human subject relies on visual information for maintaining stability, the greater are the increases in the levels of neurological activity in the prefrontal lobe, visual association cortex, and parietal lobe; this because there is a need to increase the processing of the postural stability information and to upgrade sensorimotor integration. In parallel, the LF group of elderly individuals demonstrated significant and strong correlation between posture-related cortical regions for the sensorimotor integration; however, the HF group of elderly individuals did not show such a relationship. Moreover, we have clarified the roles of brainwave bands in motor control, and it was found that the gamma and beta bands in the parietal–occipital region facilitate high-level cortical modulation and sensorimotor integration, whereas the theta band in the frontal–central region is responsible for mediating error detection during perceptual motor tasks. Finally, the alpha band within the occipital lobe is associated with processing visual challenges. With motor control demands varying over time, increments in brainwave band coordination would seem to be required for the maintenance of postural stability.

The comprehensive investigations used in this study have shed light on cortical modulation during motor control among elderly participants with varying level of potential risk of falling. The fact that there is a fundamental neural effect of AR suggests that this system may be a novel approach for identifying community-dwelling elderly individuals who are at increased risk of falling. Furthermore, this approach could also be used to enhance cortical modulation of neurological patients and elderly individuals who are at an increased risk of falling when in challenging postures. The AR effect proves that when inducing multiple-sensory integrated modulation among the elderly, an upright position combined with the visual-motor integration training is recommended. Our results also suggest that, although the elderly adults are without neurological deficits, inefficient centrally modulation during a challenged postural condition might be an internal factor that contributes to the risk of falling. Finally, training to bring about coordinated sensorimotor integration might be a useful way of reducing the risk of fall among elderly individuals.

## Author Contributions

C-JC contributed to the study design, experiment setup, data acquisition, statistical analysis, and manuscript drafting. T-FY contributed to participant recruitment, the conception of this study, and manuscript drafting. S-WY contributed in the interpretation of data, the critical revising of the work, and the final approval of the published version. J-SC contributed to data interpretation, manuscript revision and final approval of the published version. All of the authors agree to be accountable for all aspects of the work and ensure that questions related to the accuracy or integrity of any part of the work have been appropriately investigated and resolved.

## Conflict of Interest Statement

The authors declare that the research was conducted in the absence of any commercial or financial relationships that could be construed as a potential conflict of interest.
