# Supplementary material for: Cortical Modulation of Motor Control Biofeedback among the Elderly with High Fall Risk during a Posture Perturbation Task with Augmented Reality
Source: Front Aging Neurosci. 2016 Apr 28;8:80. doi: 10.3389/fnagi.2016.00080 (PMC4848299; doi:10.3389/fnagi.2016.00080)
Supplement: Supplementary file 1 [file Table_1.DOC]

Supplementary Material

# Cortical modulation of motor control biofeedback among the elderly with high fall risk during a posture perturbation task with augmented reality

Chun-Ju Chang^1^, Tsui-Fen Yang^2-3^, Sai-Wei Yang^1*^, Jen-Suh Chern^4**^

***Correspondence:** Sai-Wei Yang, [swyang@ym.edu.tw](mailto:swyang@ym.edu.tw)

****Correspondence:** Jen-Suh Chern, [chern8616@gmail.com](mailto:chern8616@gmail.com)

# Supplementary table 1: The results of receiver operating characteristics curve analysis on center of pressure ellipse area in the VR-based posturography balance-maintaining movement

|  | LF | HF |
| --- | --- | --- |
| Area under the curve (AUC) | 0.750 | 0.971 |
| Sensitivity | 0.556 | 0.929 |
| Specificity | 0.857 | 0.875 |
| Cutoff point in center of pressure ellipse area (cm^2^) | 7.63 | 12.48 |
